# Reviewed article: Pereira, MIM; et al. American tegumentary leishmaniasis: epidemiological analysis and time series, Minas Gerais, Brazil, 2008-2024. Epidemiol Serv Saude. 2026:35;e20251212

**DOI:** 10.1590/S2237-96222026v35e20251212.b

**Published:** 2026-11-09

**Authors:** Pedro Arthur Araújo

**Affiliations:** 1 Ministério da Saúde, Brasília, DF, Brazil Ministério da Saúde Brasília DF Brazil

## Peer review A

### Questionnaire

Does the manuscript contain new and significant information to justify publication? Yes 

Does the Abstract (Summary) clearly and accurately describe the content of the article? Yes 

Is the problem significant and concisely stated? Yes 

Are the methods described comprehensively? Yes 

Are the interpretations and conclusions justified by the results? Yes 

Is adequate reference made to other work in the field? Yes 

### Manuscript Structure:

Length of article is: Adequate

Number of tables is: Adequate

Number of figures is: Adequate

### Rating

Interest Good

Quality Good

Originality Average

Overall Good

Recommendation: **Minor Revision**

## Comments

1 - A Introdução deve ser revisada integralmente para melhorar fluidez, precisão e correção gramatical. Trechos com estrutura inadequada, redundâncias, falta de verbos principais e repetições.

Linhas 14-16: frase incoerente e redundante (“…ampla distribuição no Brasil e no território brasileiro…”). Sugiro reescrita completa.

Linha 19: ausência de conjunção (“mantém caráter endêmico está associada”).

Linhas 17-20: falta de pontuação; inserir “e”: “mantém caráter endêmico e está associada…”.

Linhas 20-27: ideias repetidas sobre urbanização, ecossistemas e desigualdade.

Linhas 31-36: duplicação do argumento sobre flutuações temporais.

Linhas 38-46: frase longa com excesso de vírgulas; sugerir divisão em duas sentenças.

Justificativa do estudo precisa ser mais objetiva. Embora a relevância esteja implícita, falta explicitar claramente o diferencial do trabalho. Explorar mais a importância e porque de:

- série temporal longa
- importância para vigilância epidemiológica regional

Sugiro reforçar esse argumento ao final da Introdução.

Métodos carecem de pequenas explicitações que fortalecem rigor científico

- Linhas 62-65: explicar como casos duplicados/inconsistentes foram identificados.
- Linha 96: justificar exclusão da categoria “ignorado/branco”.
- Linha 86: especificar o pacote do R utilizado para o teste de Mann-Kendall.
- Variáveis de faixa etária muito segmentadas. Essa categorização foi baseada em que? Um estudo? Algum documento técnico?
- Linha 88: indicar data exata de download da base.

Resultados

- Linha 108: incluir as taxas anuais mínimas e máximas.
- Linha 119: comentar proporção relativamente alta de “indeterminado” (5,9%). E levar para discussão para ampliar os argumentos.

Discussão pode ser mais crítica e menos descritiva. Em alguns trechos, há repetição de informações dos Resultados sem aprofundar implicações epidemiológicas. Nesse sentido, deixo como sugestão:

- integrar comparação com tendências nacionais e estaduais;
- explorar implicações para vigilância, diagnóstico e controle vetorial;
- refletir sobre impacto de autóctones elevados;
- incorporar limitações do estudo (dados secundários, subnotificação, ausência de análise espacial).
- Linhas 150-154: repetição do argumento sobre irregularidade temporal.
- Linhas 162-165: esclarecer que o estudo não realizou análise espacial, embora dialogue com literatura que fez.
- Linhas 180-187: aprofundar discussão sobre formas mucosas e diagnóstico.

Quanto à redação geral do manuscrito, deixo como observações:

- Evitar repetição do termo “macrorregião norte de Minas Gerais” quando o contexto já estiver claro.
- Simplificar frases extensas.
- Padronizar termos epidemiológicos.

